# Novel Fabrication of Zein-Soluble Soybean Polysaccharide Nanocomposites Induced by Multifrequency Ultrasound, and Their Roles on Microstructure, Rheological Properties and Stability of Pickering Emulsions

**DOI:** 10.3390/gels7040166

**Published:** 2021-10-13

**Authors:** Teng Song, Zhiyu Xiong, Tong Shi, Abdul Razak Monto, Li Yuan, Ruichang Gao

**Affiliations:** 1 School of Food and Biological Engineering, Jiangsu University, Zhenjiang 212013, China; st181019@163.com (T.S.); xiongzhiyu0304@163.com (Z.X.); shi2018tong@163.com (T.S.); 5103200370@stmail.ujs.edu.cn (A.R.M.); 2 College of Life Science, Anhui Normal University, Wuhu 241000, China

**Keywords:** zein, soluble soybean polysaccharide, high internal phase emulsions, multifrequency ultrasound, stability

## Abstract

In this work, soluble soybean polysaccharides (SSPS) were employed together with multifrequency ultrasound to fabricate zein nanocomposites which were conducive to enhancing the stability of high internal phase emulsions (HIPEs). Compared with non-ultrasonic treated zein colloidal particle samples (132.23 ± 0.85 nm), the zein nanoparticles samples induced by dual-frequency ultrasound exhibited a smaller particle size (114.54 ± 0.23 nm). Furthermore, the particle size of the zein composite nanoparticles (256.5 ± 4.81) remarkably increased with SPSS coating, consequently leading to larger fluorescence intensity together with lower zeta-potential (−21.90 ± 0.46 mv) and surface hydrophobicity (4992.15 ± 37.28). Meanwhile, zein-SSPS composite nanoparticles induced by DFU showed remarkably enhanced thermal stability. Fourier transform infrared (FTIR) spectroscopy and Circular dichroism (CD) spectroscopy were also used to characterize zein-SSPS composite nanoparticles. The results confirmed that DFU combined with SSPS treatment significantly increased β-sheets (from 12.60% ± 0.25 b to 21.53% ± 0.37 c) and reduced α-helix content (34.83% ± 0.71 b to 23.86% ± 0.66 a) remarkably. Notably, HIPEs prepared from zein-SSPS nanocomposites induced by dual-frequency simultaneous ultrasound (DFU) at 40/60 kHz showed better storage stability. HIPEs stabilized by DFU induced zein-SSPS nanoparticles exhibited higher storage modulus (G′) and loss modulus (G″), leading to lower fluidity, together with better stability contributing to the water-binding capacity and three-dimensional (3D) network structure of the HIPEs emulsion. The findings of this study indicate that this method can be utilized and integrated to further extend the application of zein and SSPS and explore HIPEs.

## 1. Introduction

Pickering emulsions are stabilized by solid particles, which retain the basic properties of conventional emulsions stabilized by surfactants. In particular, the solid particles adsorb onto the oil-water interface of the emulsions, which endows these emulsions with excellent interfacial properties, thereby acting to prevent droplet aggregation. In comparison with conventional emulsions, Pickering emulsions exhibit a thicker interface, higher surface loads, and better stability [1]. In recent years, benefiting from tunable surface properties and the rapid fabrication techniques of Pickering emulsions, Pickering emulsions have attracted increasing attention in many cutting-edge fields, such as drug delivery, medical diagnosis, tissue engineering, and the preparation of novel materials.

HIPEs (High internal phase emulsions) are biphasic systems with minimal dispersed phase volume fractions exceeding 74% [2]. Low molecular weight surfactants with high concentration are usually used to stabilize traditional HIPEs. Recently, their applications in food formulations were realized. Unfortunately, many of the model systems have been fabricated based on inorganic or synthetic polymer-based surfactants. Therefore, the large-scale use of surfactants in industrial applications is not cost effective, and in some cases may cause the potential safety and environmental issues for the emulsion systems. Therefore, their applications are limited in the food industry. As a substitute for low molecule weight surfactants constructed conventional HIPEs, Pickering HIPEs, which are stabilized by solid particles, have attracted tremendous attention in recent decades [3] due to their higher resistance to coalescence and Ostwald ripening, environmental friendliness, and low toxicity [4]. To date, research on Pickering emulsions stabilized by solids mainly focuses on inorganic or synthetic particles, such as silica [5], titanium dioxide [6], saponite clay [7], and zinc oxide [8]. Although the Pickering emulsions prepared by inorganic particles have good stability, most of them are not food grade and their biocompatibility is poor. Moreover, more attention should be paid to their processing techniques which inevitably result in high cost, low yield, complex craftwork and serious waste pollution, etc. Currently, seeking environmentally friendly and natural sources as well as renewable and degradable biological particles to stabilize Pickering emulsions has become a hot spot of research, particularly in foods, pharmaceuticals and cosmetics.

Nowadays, a wide variety of bioderived polymers, such as proteins, carbohydrates, and fats, have been investigated to fabricate Pickering emulsions. Protein-based particles in Pickering emulsions’ stability were realized, possibly attributed to the many promising physicochemical properties and functional groups, which endows the protein particles with a strong ability to adsorb onto the oil-water interface. Protein particles can be derived from various biomass sources, including soy protein isolate [9], pea protein isolate [10], gliadin [11], wheat protein isolate [12], and zein [2]. As a natural protein, zein contains both hydrophilic and lipophilic groups, which means that it has several attractive features, such as good biocompatibility and adhesion properties. The hydrophobic region of zein can be polymerized into colloidal particles, leading to zein being the most commonly used protein in emulsions [13]. However, Pickering emulsions stabilized merely by zein are prone to delamination, demulsification, oil leakage, etc. [14]. Therefore, the modification of zein to improve the stability of zein-stabilized Pickering emulsions is warranted.

In recent years, many attempts have been made to explore the synergistic effects of zein and other biological substances on the stability of emulsions, such as polyphenols [11], proteins [15], polysaccharides [16]. Protein–polysaccharide complexes formed via electrostatic interaction due to electrosteric repulsion and increasing the viscosity can be used for emulsion stabilization [17,18,19]. Soluble soybean polysaccharide (SSPS), which was isolated from the soybean slag made from soy protein isolate, soy milk, tofu, and fermented soybean products [20], is a negative polysaccharide whose molecular structure resembles a spherical shape [21]. SSPS is normally used as a stabilizer to prevent acidic protein beverages from forming precipitation [22]. It has been suggested that SSPS could be used in flavor emulsions because of its high water solubility, low bulk viscosity, high temperature stability, emulsifying properties and its ability to form strong interfacial films [23]. A previous report have shown that the stability of emulsions could be improved with the addition of SSPS via steric repulsion [24]. Li and Wang reported that SSPS could adsorb on the surface of zein colloidal particles in a wide pH range due to electrostatic and hydrophobic interactions, thus contributing to the stability of zein colloidal particles in an aqueous solution [25]. Gao et al. studied the effects of zein and SSPS concentration on the properties of zein-SSPS colloidal particles and the emulsions, indicating that the Pickering emulsions exhibited higher stability at 25 °C at zein concentration of 6 mg/mL and SSPS concentration of 1 mg/mL, which was because of the densely packed interface layer preventing oil droplets from coalescing and Ostwald ripening [26].

Many auxiliary methods have been used to assist in the preparation of stabilizers for Pickering emulsions, including microfluidics [27], chemical crosslinkers [28], and ultrasound [15]. As an emerging non-thermal processing physical technology, multifrequency ultrasound treatment has been applied to zein modification, contributing to structural and physical changes in zein [29]. Furthermore, multifrequency ultrasound treatment can change the electrostatic and hydrophobic interactions between zein and chitosan, which enhance the encapsulation efficiency (EE), the loading capacity (LC) of resveratrol and improves the thermal stability of the zein-chitosan complex [20]. Presumably, ultrasound treatment might improve the thermal stability of zein-SSPS colloidal particles. However, there are few reports about the combined effects of frequency ultrasound and SSPS on zein-based colloidal particles and HIPE Pickering emulsions.

Hence, the effects of different multimodel frequency ultrasound treatments on the preparation and properties of the zein-SSPS complex were investigated in this study. Furthermore, the zein-SSPS complex was used to prepare a HIPE Pickering emulsion, and properties of the HIPE Pickering emulsion were studied subsequently.

Therefore, the main objective of this work was to explore the promising potential of nanoparticles fabricated from zein and SSPS in food-grade Pickering emulsion stabilization. First, zein and SSPS dispersions were subjected to either intact or thermal treatments. The effects of DFU and SSPS treatments on the interfacial properties of zein particles were then investigated. Furthermore, the potential application of these nanoparticles in Pickering stabilization was monitored and compared with that of zein particles subjected to similar treatments.

## 2. Results and Discussion

### 2.1. Effect of Ultrasonic Modes on Storage Stability of HIPEs Stabilized by Zein Composite Nanoparticles

**Figure 1:**
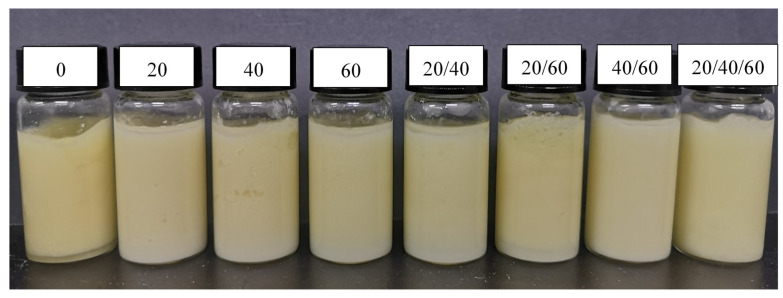
The appearance of Pickering emulsions stored for 30 days.

Storage stability is an important indicator for judging the quality of the emulsion. As shown in Figure 1, the HIPEs, which were stabilized by zein treated with different ultrasonic modes (SFU, DFU, MFU), had been stored for 30 days at 4 °C, and the prepared Pickering emulsions were summarized in Table 1. The HIPEs treated without frequency ultrasound, SFU, and DFU (20/40, 20/60 KHz) appeared stratified and underwent oil leak and collapse, indicating that these HIPEs samples were unstable systems. For the same treatment time, the stability of the Pickering emulsion stabilized by zein gradually increased with the increase in the ultrasonic frequency. Additionally, when the two frequencies worked together, the stability gradually increased with the increase in the single frequency. However, when the three frequencies were used in combination, the stability of the Pickering emulsion decreased. Furthermore, no similar phenomenon appeared for the HIPEs stabilized by zein treated with DFU (40/60 KHz). The zein treated with DFU (40/60 kHz) exhibited the best potentiality to stabilize HIPEs. It is speculated that the ultrasonic frequency at 40 kHz was close to the inherent frequency of zein, achieving the optimum coupling [29]. With the increase and superposition of ultrasonic frequency, more hydrophobic groups of zein leaked out, resulting in the adsorption of zein particles at the oil–water interface and the formation of more stable oil–water colloidal particles. However, when the three ultrasonic frequencies were superimposed, the strong ultrasonic mechanical and cavitation effect caused the aggregation of protein colloidal particles, and then the adsorption of zein particles at oil–water interface decreased, resulting in the decrease in emulsion stability. Thus, the DFU (40/60 kHz) model was selected for subsequent experimental studies.

### 2.2. Physicochemical Properties of Zein Composite Nanoparticles

#### 2.2.1. Zeta-Potential, PDI and Particle Size Distribution of Nanoparticles

**Figure 2:**
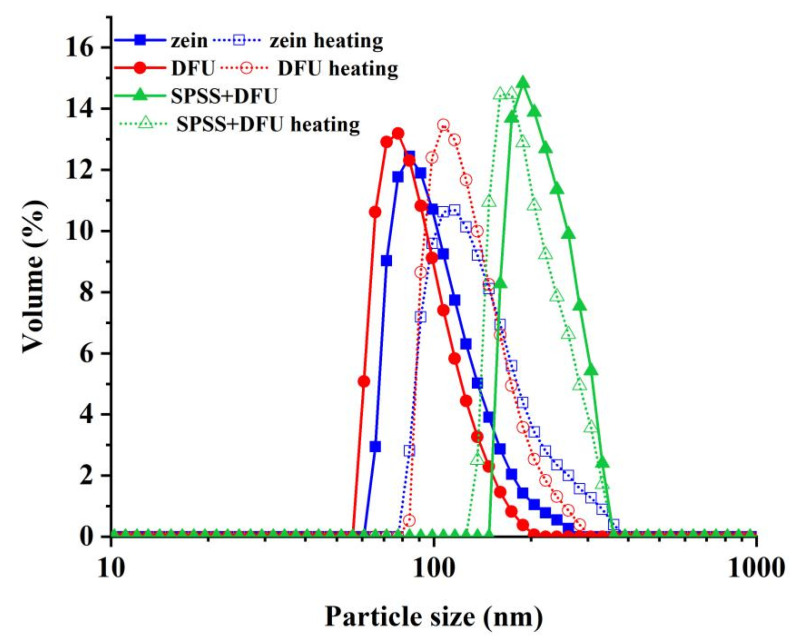
The particle size of colloidal particles: zein: the colloidal particles prepared with native zein; zein + DFU: the colloidal particles prepared with zein treated with DFU; zein + DFU + SSPS: the colloidal particles prepared with DFU combined with SSPS. (The solid curves represent the particle size of colloid particles without heating treatment; the modest curves represented the particle size of the colloid particles heated for 60 min).

The size of the colloidal particles affects the stability of the suspension and the emulsion formed by the colloidal particles [30]. As shown in Figure 2, the particle size of zein colloidal particles was less than 300 nm. For zein colloidal particles treated with DFU, the particle size was below 200 nm which was less than the particle size of the zein colloidal particle. The decrease in the size of the particles treated with DFUTreated with DFU, the particle size decreased, indicating that dual-frequency simultaneous ultrasound caused compact zein particles to disperse into small particles. According to a previous study [31], the effect of ultrasound on particle size can be attributed to the ultrasonic cavitation bubbles generated in the suspension. Moreover, the thermal and non-thermal effects of ultrasound caused a sharp increase in temperature and pressure around the ultrasonic cavitation bubble, which broke protein aggregates, resulting in a decrease in particle size. The result agreed with the changes in the hydrodynamic radius (shown in Table 2). However, once zein was combined with soluble soybean polysaccharide, the particle size increased sharply (most of the particles about 200 nm in size, and the largest particle was about 400 nm), indicating that zein interact with soluble soybean polysaccharide to form a complex with larger particle size in the form of a covalent bond or non-covalent bond.

The zeta-potential of native zein colloidal particles was found to be positively charged with +18.7 mV and became highly negative (−21.90 mV) after zein particles were coated with soluble soybean polysaccharide. Similar results were also observed in other studies, which confirmed that soluble soybean polysaccharide was successfully coated on the surface of zein nanoparticles by electrostatic interactions [20,32]. However, DFU had no significant (*p* > 0.05) effect on the zeta-potential of the zein colloidal particle. It could be concluded that the surface charge of the zein colloidal particle was not changed upon DFU treatment, even though this treatment significantly decreased the particle size of the zein colloidal particle. Furthermore, DFU treatment decreased the PDI of the zein colloidal particle from 17.47% to 11.30%, indicating that the complex became more uniform owing to ultrasonic cavitation.

#### 2.2.2. Atomic Force Microscopy (AFM)

**Figure 3:**
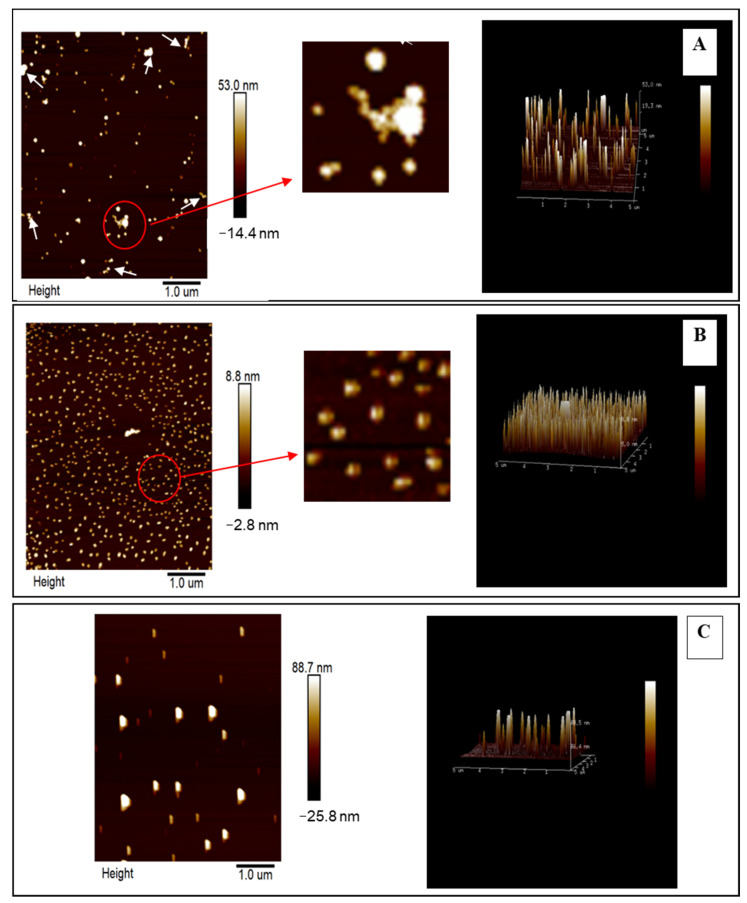
Atomic force microscopy of native zein colloidal particles (**A**), zein colloidal particles treated with DFU (**B**) and zein colloidal particles treated with DFU combined with SSPS (**C**).

Atomic force microscopy (AFM) provides an alternative to image and manipulate single biological macromolecules with high resolution [33]. Figure 3A shows that the particle size distribution of native zein colloid particles was uneven. It was also found that the behavior of several different colloids aggregated in Figure 3A. After ultrasonication (Figure 3B), the particle size distribution and shape of zein colloid was uniform, and no aggregates were observed due to ultrasound treatment. This suggests that DFU is effective in producing zein colloidal particles. It is speculated that disaggregation of zein aggregates occurred, contributing to the cavitation caused by DFU, which could reduce the particle size of zein colloidal particles. After being coated with SSPS, zein-coated SSPS colloid particles exhibited the increasing particle size, which was even larger than that of zein. This suggests that polymers with larger particle size were formed in colloidal suspensions due to the combination of zein and SSPS by the application of DFU. Moreover, a data analysis based on AFM further confirmed the significant increase in aggregation with increases in the particle sizes of the zein-SSPS nanoparticles. The results were also consistent with the change of polydispersity index, and hydrodynamic diameter (Table 1). Similar results [34,35] have been found in previous reports which indicated the stabilization of Pickering emulsions via protein isolate nanoparticle aggregates. Therefore, SSPS used to glycate zein to form nanoparticles can be used to control interfacial activities and emulsion stabilization.

#### 2.2.3. Fluorescence Spectroscopy and Surface Hydrophobicity

**Figure 4:**
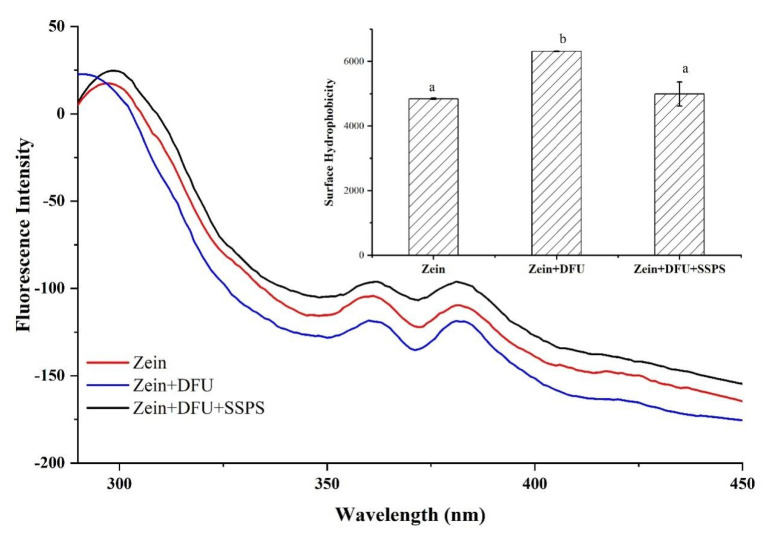
Fluorescence spectroscopy spectra and surface hydrophobicity of different samples: zein: the colloidal particles prepared with native zein; zein + DFU: the colloidal particles prepared with zein treated with DFU; zein + DFU + SSPS: the colloidal particles prepared with DFU combined with SSPS. Different letters (a–b) on the same column are significantly different among the different samples (*p* < 0.05). Each treatment was performed in triplicate (n = 3).

The intrinsic fluorescence property is usually used to evaluate the interaction between proteins and polysaccharides, which is mainly contributed by aromatic amino acids such as tryptophan and tyrosine. The fluorescence emission spectroscopy of the samples was evaluated at the excitation wavelength and emission wavelength 280 nm and 290–450 nm, respectively. As shown in Figure 4, the intrinsic fluorescence peak of the native zein was around 300 nm, which agreed with a previous study [36]. For the samples treated with DFU, the emission fluorescence spectrawas blue shifted, indicating that polarity of the environment surrounding tryptophan residues was reduced and a protein folding/unfolding process occurred due to DFU [20], resulting in the shifting of more hydrophobic clusters in the interior of the molecule to protein surface [37] and an increase in surface hydrophobicity. However, once combined with SSPS, the intrinsic fluorescence peak of zein did not change significantly, whereas the fluorescence intensity increased gradually. The hydrophobic interactions of zein molecules were destroyed due to DFU treatment, which promoted more hydrophobic groups to be exposed to the surface due to the cavitation, microstreaming and turbulent forces of the ultrasound treatment and the interaction between zein and SSPS, increasing fluorescence intensity [38,39].

#### 2.2.4. Fourier Transform Infrared (FTIR) Spectroscopy

**Figure 5:**
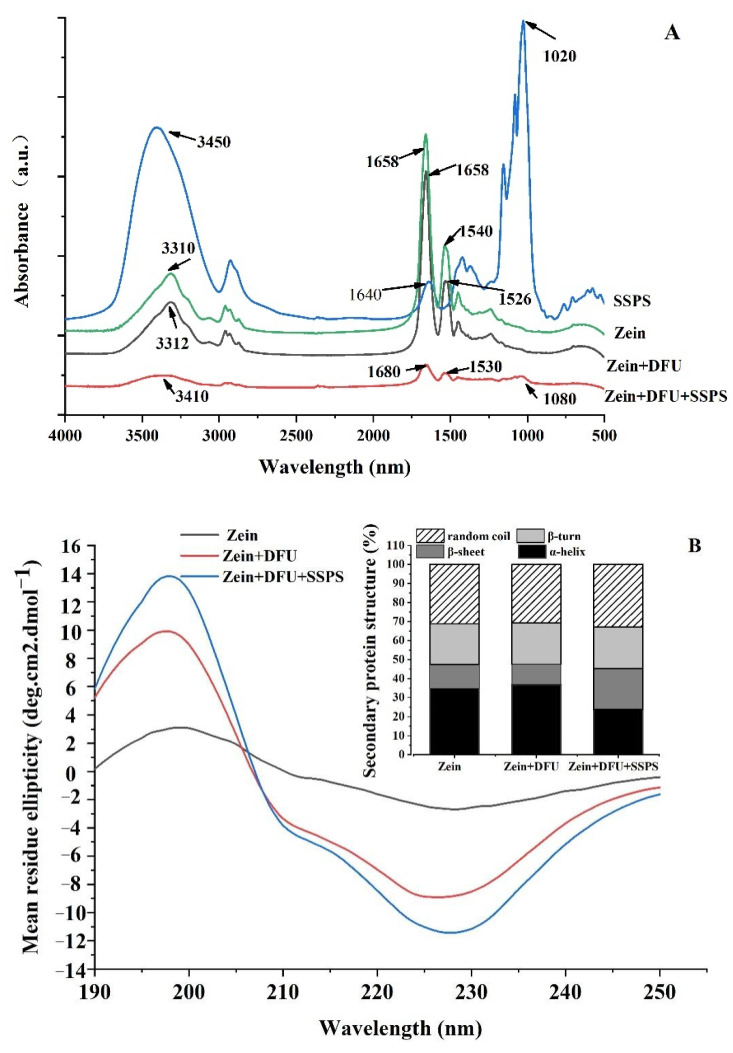
Fourier transform infrared spectroscopy spectra (**A**) and CD spectroscopy spectra (**B**) of different samples: zein: the colloidal particles prepared with native zein; zein + DFU: the colloidal particles prepared with zein treated with DFU; zein + DFU + SSPS: the colloidal particles prepared with DFU combined with SSPS.

FTIR is a common way to analyze the secondary structure of organic matter. As shown in Figure 5A, native zein showed a prominent absorption peak at 3310 cm^−1^, caused by the stretching vibration of N–H [40]. Compared to native zein, zein treated with DFU shift little at the hydrogen bond (3200–3400 cm^−1^, N–H stretching) position, revealing that DFU could not destroy hydrogen bond of zein. For the samples of zein coated with SSPS under ultrasonication, the hydrogen bond positions shifted from 3310 cm^−1^ to 3410 cm^−1^, indicating that zein and SSPS interacted with each other by hydrogen bonds, facilitating the formation of new complex. In addition, amide II band (1530–1550 cm^−1^, N–H deformation, and C–N stretching) positions shifted from 1540 cm^−1^ to 1526 cm^−1^ and 1530 cm^−1^, respectively. These results indicated that the electrostatic interactions between zein and SSPS treated with DFU. Furthermore, 1700–1600 cm^−1^ is the peak position of the amide I band and the peak position of the α-helix is 1650–1660 cm^−1^. After zein was coated with SSPS under ultrasonication, the peak position shifted from 1658 cm^−1^ to 1680 cm^−1^, indicating that the addition of SSPS changed the secondary structure of zein. Moreover, after adding SSPS to the zein suspension, a new peak appeared at 1080 cm^−1^, which might be attributed to the combination of zein and SSPS leading to the formation of a stable complex.

#### 2.2.5. Circular Dichroism (CD) Spectroscopy Analysis

CD spectroscopy is a technical method to used evaluate protein conformation in dilute solutions [41]. As shown in Figure 5B, the CD spectrum of zein indicated that zein possessed a typical α-helical-rich secondary structure with a positive peak at 198 nm and two negative peaks at 203 nm and 226 nm, which agreed with the previous study [42]. After treating with DFU, the secondary structure of zein did not change distinctly (α-helix shifted from 34.8% to 36.7%, β-sheet shifted from 12.6% to 10.9%, β-turn shifted from 21.4% to 21.6%, random coil shifted from 31.2% to 30.9%), indicating that DFU treatment did not change the secondary structure of the protein. When SSPS was added, it was observed that β-sheet increased significantly from 12.6% to 21.5%, whereas α-helix significantly decreased from 34.8% to 23.8%. It was obvious that the increase in β-sheet content was at the expense of the decrease in α-helix content during the formation of colloidal particles. It indicated that the addition of SSPS significantly altered the secondary structure of the zein leading to protein unfolding and restructuring, and finally forming a compact structure [43].

#### 2.2.6. Thermal Stability

Long-term heat treatment induced protein aggregation. As the heating time and temperature increased, the larger colloidal particles formed [29]. As shown in Figure 2, the hydrodynamic diameter of the zein colloidal particle was 177.20 nm after heat treatment, which was 31.02% larger than the hydrodynamic diameter of unheated zein colloidal particles, and the hydrodynamic diameter of zein treated with DFU was 156.30 nm, which was higher by 36.46% than that of the unheated sample. The hydrodynamic diameter of zein-SSPS complex colloidal particles treated with DFU was 252.94 nm, which was an increase of 1.39% compared with the unheated sample, and the change was not significant. Compared with free SSPS colloidal particles, zein-SSPS complex particles exhibited better thermal stability. The improved thermal stability was possibly ascribed to the steric hindrance, which prevented the aggregation of colloidal particles [44,45].

### 2.3. Rheological Properties, Microstructure and Stability of HIPEs

#### 2.3.1. Rheological Properties of Emulsions Stabilized by Zein-SSPS Composite Nanoparticles

**Figure 6:**
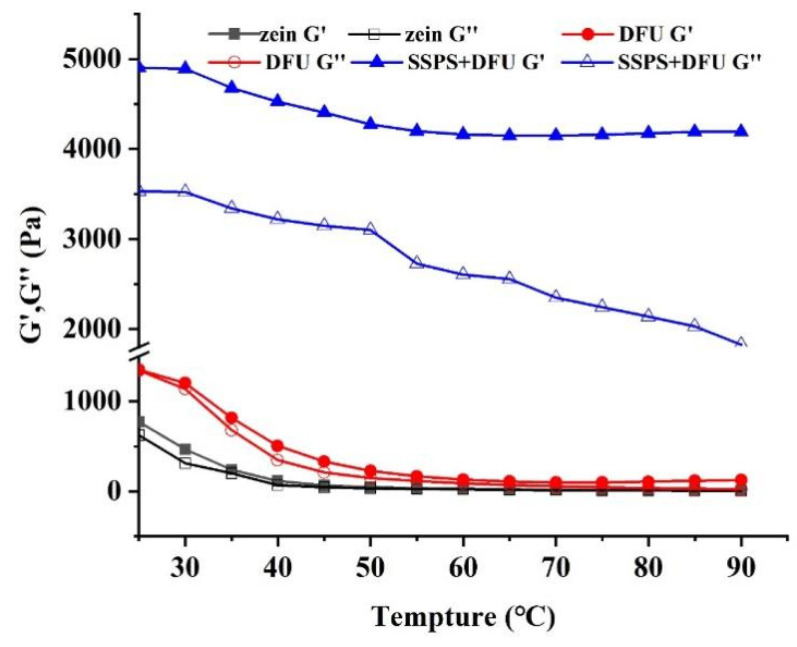
The changes of G′ (filled symbols) and G″ (open symbols) of HIPEs during temperature scanning. zein: the colloidal particles prepared with native zein; zein + DFU: the colloidal particles prepared with zein treated with DFU; zein + DFU + SSPS: the colloidal particles prepared with DFU combined with SSPS.

Rheological properties are of great significance to the stability of the emulsions. The strength of the emulsion interface film can be reflected by the interface modulus (G′ and G″) and result in the stability of the emulsion. Compact interface film and great mechanical strength of emulsions could decrease the probability of collision, which is necessary to prevent the accumulation of oil droplets, thereby stabilizing the emulsion. As shown in Figure 6, the storage modulus (G′) of all samples was greater than loss modulus (G″), indicating that all HIPEs stabilized by zein colloidal particles exhibited solidlike properties. The moduli of HIPEs stabilized by zein treated with DFU were greater than those of HIPE stabilized by native zein, which could be ascribed to the smaller particle size of zein treated with DFU. Smaller particle size is more advantageous in the stabilization of Pickering emulsions. Indeed, the smaller the particle size, the more particles adsorbed at the oil–water interface, which is beneficial for stabilizing the higher interface area and thicker interfacial layer [46]. After thermal treatment, the moduli of the HIPEs stabilized by native zein and zein treated with DFU went down significantly, even approaching zero after heating, indicating that the these HIPEs samples had poor resistance to thermal aggregation. The moduli of HIPE stabilized by zein treated with DFU combined with SSPS increased evidently, which might be attributed to the three-dimensional network structure formed by zein coated SSPS enhancing the strength of the emulsion gel [47]. After thermal treatment, the moduli did not change significantly, indicating that HIPEs stabilized by zein treated with DFU combined with SSPS had a better ability to resist heat accumulation and exhibited better thermal stability.

#### 2.3.2. Microstructure of Emulsions Stabilized by Zein-SSPS Composite Nanoparticles

**Figure 7:**
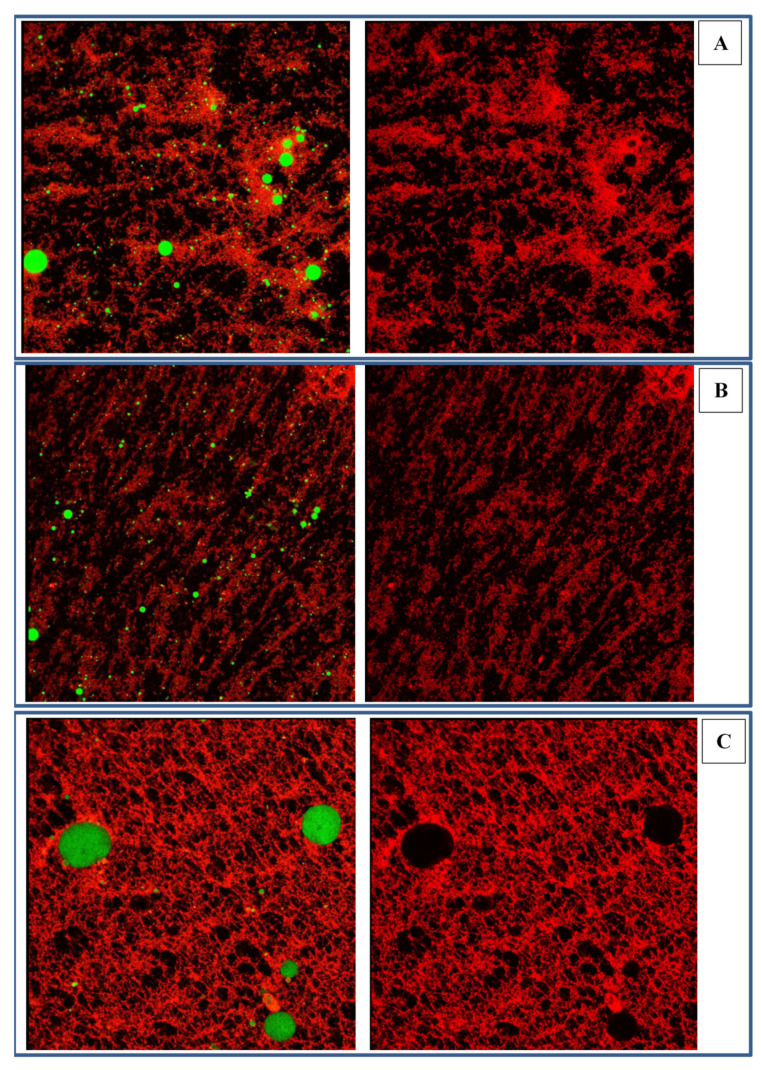
The CLSM images of Pickering emulsion ((**A**). Emulsion stabilized by native zein colloidal particles; (**B**). Emulsion stabilized by zein colloidal particles treated with DFU; (**C**). Emulsion stabilized by zein colloidal particles treated with DFU combined with SSPS).

Confocal Laser Scanning Microscopy (CLSM) is usually used to observe the distribution of oil droplets and proteins in the emulsion. Figure 7 is a CLSM diagram, in which the red part was stained with Fast Green, representing the distribution of the proteins. Meanwhile, the green part was stained with Nile red, representing the distribution of the oil droplets. As shown in Figure 7A, the oil droplets stabilized by native zein colloidal particles were small, and the protein distribution was uniform. Upon further observation, there was almost no protein aggregation on the surface of the oil droplets. When treated with DFU (Figure 7B), the oil droplets were smaller than those stabilized by native zein, contributing to the enhancement of stability. As shown in Figure 7C, a three-dimensional (3D) network structure was formed once zein was combined with SSPS, which fixed the oil droplets to prevent them from aggregating. It follows from the above that appropriate use of SSPS could improve the stability of the emulsion. For Figure 7C, lots of network structures were available without oil droplets illustrated that the visible network structure and the oil drops may not on the same plane. It is speculated that DFU treatment causes zein to expose more hydrophobic groups [48], which allows zein to provide more binding sites and bind more SSPS. Moreover, SSPS molecules also bind to each other through non-covalent bonds. On this basis, a three-dimensional spatial structure is formed due to molecular cross-linking [49]. Owing to the formation of the three-dimensional network structure, the oil droplets were firmly anchored in a three-dimensional network, less likely to collide with each other, so that the emulsion remained stable even though the oil drops were larger.

#### 2.3.3. Stability of Zein-SSPS Stabilized HIPEs Based on Water Migration

**Figure 8:**
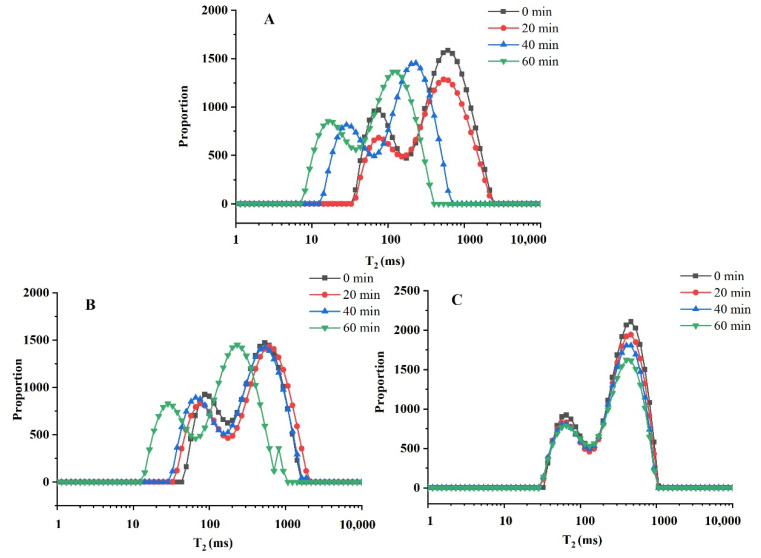
The distribution of transverse relaxation time (T_2_) spectra of different samples: Pickering emulsion stabilized by native zein colloidal particles (**A**), Pickering emulsion stabilized by zein colloidal particles treated with DFU (**B**) and Pickering emulsion stabilized by zein colloidal particles treated with DFU combined with SSPS (**C**).

As a non-destructive testing technology, LF-NMR has been more and more widely used in the food industry [50]. As for the characterization of emulsions, LF-NMR has been used to characterize the emulsion transformation process in addition to the conventional characterization index [51]. The value of the relaxation time T_2_ can reflect the degree of freedom of moisture in emulsions, and the change of T_2_ characterizes the distribution and mobility of various states of moisture in emulsions after heating treatment for different times, that is, the binding state and free movement of moisture in each state. Furthermore, the relaxation peak area percentage can estimate the relative content of hydrogen protons, thereby reflecting the content of various states of water groups, and its changes can characterize the changes in the content of various states of water molecules in emulsions after heating treatments for different times. As described in the precious reference, the distribution of the relaxation time T_2_ presented three peaks: 0–10, 10–100, and 100–1000 ms. According to the peak time and the percentage of the total area occupied by each peak, the three peaks were considered to correspond to the three components of water, namely bound water (T_2b_), non-flowing water (T_21_), and free water (T_22_) [50]. As shown in Figure 8A, the T2 distribution of the emulsions heated for 20 min did not change significantly compared with fresh emulsion, and the hydrogen proton intensity was slightly reduced, indicating that short-term heat treatment did not cause changes in water holding capacity of the proteins. Furthermore, it was also found that prolonging the heating time was accompanied by left shifts of the T2 distribution of the emulsions stabilized by zein, indicating that the heating treatment caused denaturation of the protein and enhanced its water holding capacity. The T2 distribution (Figure 8B) of the emulsions stabilized by zein treated with DFU did not change distinctly after heated for 20 and 40 min, which indicated that the emulsions stabilized by zein treated by DFU had better water holding capacity compared with zein-stabilized emulsions. With the extension of the heating time, the T2 distribution shifted to the left, indicating that the prolonged heating denatured the protein and strengthened the water holding capacity. Due to ultrasonic cavitation, the size of protein particles reduced, and the cross-links between proteins increased, which facilitated the penetration and filling of small molecules such as water molecules, allowing free water to fill around the amino acid side chains of the protein to increase water holding capacity [52]. However, the T_2_ of emulsion stabilized by zein treated with DFU combined SSPS (Figure 8C) did not change, both in peak position and peak area, indicating that zein treated with DFU combined SSPS exhibited good resistance to thermal accumulation.

## 3. Conclusions

The HIPE Pickering emulsions were fabricated using zein and SSPS induced by DFU. The zein treated with DFU at 40/60 kHz contributed to great storage stability, following the ultrasonic frequency at 20/40/60 kHz. When treated with DFU, the size of the zein colloidal particles decreased significantly, and the fluorescence intensity and surface hydrophobicity increased; however, the zeta-potential did not exhibit any significant change. Based upon coating with SSPS, the particle size and fluorescence intensity of zein composites nanoparticles were obviously increased. Moreover, the zeta-potential and surface hydrophobicity were relatively decreased compared with colloidal particles treated with DFU. FTIR and CD spectroscopy were further used to characterize the secondary structure of zein, revealing that DFU did not change the secondary structure unless zein was coated with SSPS. Furthermore, the thermal stability of zein colloidal particles was obviously improved after DFU treatment and coating with SSPS. The rheological properties of the HIPE Pickering emulsions stabilized by zein-SSPS composite nanoparticles induced by DFU were improved. By means of LF-NMR analysis, it was proved that the water-binding capacity of zein colloidal particles was improved upon coating with SSPS. Using CLSM, the formation of a three-dimensional network structure in emulsions stabilized by zein-SSPS composite nanoparticles was confirmed, resulting in an improvement in the stability of the Pickering emulsion. Thus, this study reveals that DFU and SSPS treatment can be employed to design and fabricate high stability HIPE Pickering emulsions in the food industry.

## 4. Materials and Methods

### 4.1. Materials

Zein (protein content of 90%) was purchased from Sigma-Aldrich, Steinheim, Germany. Soluble soybean polysaccharide (SSPS) was provided by Henan Wan Bang Industrial Co., Ltd. (Zhengzhou, China). Soybean oil was produced by Yihai Kerry Arawana Holdings Co., Ltd. and purchased from a local supermarket (Zhenjiang Kaiyuan Tourist Supermarket, Zhenjiang, China). Other reagents used were of analytical grade and were purchased from Sinopharm Chemical Reagent Co., Ltd. (Shanghai, China).

### 4.2. Preparation of Zein-SSPS Colloidal Particles with Different Model Frequency Ultrasound

The ultrasound equipment with different model frequencies was developed by a research team at Jiangsu University and manufactured by a company named Meibo Biotechnology Co. (Zhenjiang, Jiangsu, China). The instrument is equipped with three frequency generators (20, 40, and 60 kHz) that can work sequentially or simultaneously. The maximum output power of the generators is 300 W and the maximum capacity of this instrument is 3L. Samples with zein concentration of 3% (*w*/*v*) were kept in the valve bags and treated with three different frequency models: single-frequency ultrasound (SFU) with frequency of 20, 40, 60 kHz, dual-frequency simultaneous ultrasound (DFU) with frequency of 20/40, 20/60, and 40/60 kHz, and multifrequency simultaneous ultrasound (MFU) with frequency of 20/28/40 kHz. The ultrasound time was 40 min and the pulsed on/off time was 10 s/3 s. The temperature of 25 ± 2 °C was maintained by placing the samples in a thermostatic water bath. After ultrasonication, the samples were dropped into 3 times volumes of SSPS solution a zein/ssps ratio of 1:0.4 (*w*/*w*). A magnetic stirrer (HJ-2A, Jiangsu Jinyi Instrument Technology Co., Ltd., Jiangsu, China) was used to ensure the mixer was fully dispersed. As control, native zein and zein treated with DFU was dropped 3 times into water under magnetic stirring The resulting suspension was rotationally steamed in a water bath at 45 °C to remove some of the water and ethanol so that colloidal suspension with zein concentration of 3% (*w*/*v*) was obtained. The colloidal solids and emulsions prepared with native zein were marked as native zein in the figures, the colloidal solids and emulsions prepared with zein treated with DFU were marked as DFU, and the colloidal solids and emulsions prepared with zein treated with DFU combined with SSPS were marked as DFU+SSPS. Finally, part of the obtained composite colloidal particle solution was stored in 4 °C refrigerators, and the other part was freeze-dried for further use.

### 4.3. Preparation of High Internal Phase Pickering Emulsion (HIPE)

The HIPEs was prepared according to a previous report with slight modifications [53]. The preceding zein and zein-SSPS particles suspensions were used to prepare Pickering emulsions. All emulsions were prepared with an oil–water ratio of 75:25 (*v*/*v*). Briefly, 7.5 mL of soybean oil was added to 2.5 mL of colloidal particle suspension in a sample vial with a maximum capacity of 15 mL. Then, the mixtures were sheared using a digital display high-speed dispersive homogenizer (FT-200-SH, Specimen and Model Factory, Shanghai, China) at 10,000 rpm for 2 min. The freshly prepared emulsions were stored in refrigerator at 4 °C.

### 4.4. Characteristics of Zein-SSPS Complexes

#### 4.4.1. Determination of Particle Size, Polydispersity Index (PDI) and Zeta-Potential

Particle size (average diameter), PDI, and zeta-potential were determined according to the previous method with slight modifications [54]. In brief, 0.1 mL of colloidal particle suspension was added to 9.9 mL of distilled water, then the mixture was placed in a cuvette and determined with a laser particle size analyzer (Mastersizer 3000, Malvern Instruments, Worcestershire, UK). All measurements were performed at 25 °C and repeated three times.

#### 4.4.2. Atomic Force Microscopy (AFM)

The emulsions were assessed with a AFM (Nano ScopeIIIa, Bruker Daltonics Inc., New York, NY, USA) according to [55] with slight modifications. In brief, the colloidal particles suspension was diluted to a concentration of zein 20 μg/mL, then 10 μL of the diluted colloidal particles suspension was spread evenly on freshly cleaved mica sheets mounted on sample disks. Subsequently, the samples were dried with high purity nitrogen before imaging. Images were scanned in tapping mode in air with a scanning range of 10 μm × 10 μm. The images were processed using a software named NanoScope Analysis 2.0.

#### 4.4.3. Fluorescence Spectroscopy and Surface Hydrophobicity

The measurements of fluorescence spectroscopy and surface hydrophobicity were performed on a fluorescence spectrophotometer (Model Cary Eclipse, Varian Inc., Palo Alto, CA, USA) referring to the reported methods [20,41]. For fluorescence spectroscopy, the excitation wavelength and emission wavelength were set 280 nm and 290–450 nm, respectively (slit widths = 5 nm). ANS was used as fluorescent probe to determine surface hydrophobicity of colloidal particle suspension. In brief, colloidal particle suspensions were diluted to 0.075, 0.15, 0.3, 0.6, 1.2 mg/mL with distilled water. Subsequently, 4 mL of diluted protein suspension with 20 μL of 8 mM ANS was used for the analysis of surface hydrophobicity. The fluorescence intensity was measured at the emission wavelength and excitation wavelength, respectively, at 470 nm and 390 nm, and the slit widths were 5 nm each. We used 4 mL of distilled water with 20 μL of ANS in the absence of colloidal particle suspension as blank. The slope of the linear plot of net fluorescence values versus protein concentrations was used as an index of the protein surface hydrophobicity.

#### 4.4.4. Fourier Transform Infrared (FTIR) Spectroscopy

The FTIR spectra were obtained using a Fourier transform infrared spectroscopy (Nicolet iS10, Thermo-Scientific, Jasco Inc., Easton, MO, USA) with the wavelength of 400–4000 cm^−1^ in 64 scans. The atmosphere was used as a baseline. Briefly, about 2 mg of samples were weighed, and an 200 mg of KBr was added under the infrared lamp. After grinding for 5 min, the tablet (with a diameter of 1.3 mm) was pressed, and a full-band (500–4000 cm^−1^) scan was performed using attenuated total reflection (ATR) cell (Jasco Inc., Easton, MO, USA). Subsequently, the spectra were collected using a software named Omnic.

#### 4.4.5. Circular Dichroism (CD) Spectroscopy

Far-UV CD spectra between190 and 260 nm were monitored with a CD spectropolarimeter (Pistarp-180, Applied Photophysics Ltd. Surrey, UK) as described in a previous study [42]. The colloidal particles suspension was diluted to a concentration of 0.2 mg/mL, which was placed in a transparent cuvette with an optical length of 1 cm. The data were analyzed with a software named CDPro 1.

#### 4.4.6. Thermal Stability

The thermal stability of zein-SSPS colloidal particles was evaluated according to a previous study [28] with some modifications. In brief, the fresh zein-SSPS colloidal particle suspension was heated at 90 °C (water bath) for 60 min. After cooling to room temperature, the particle size of the colloidal particles was investigated using the method described in Section 4.4.1.

### 4.5. Properties of HIPEs

#### 4.5.1. Rheological Properties

The rheological properties of the HIPEs were investigated with a rheometer (DHR-2, TA Instruments, New Castle, DE, USA) according to a previous study [28]. A plate radius of 40 mm was used, and the linear viscoelastic range of each sample was determined in a stress range of 0.01–100 Pa at a fixed frequency of 1 Hz. The temperature sweep test was performed at a fixed frequency (1 Hz) with a temperature rising stage from 25 °C to 90 °C at the rate of 5 °C/min. To prevent water evaporation, a layer of silicone oil was covered around the samples.

#### 4.5.2. Analysis of Water Migration

Low-field nuclear magnetic resonance (LF-NMR) was used to analyze the water migration of the HIPEs during heat treatment using an LF-NMR analyzer (NM120-030V-I, Niumag Co., Ltd., Suzhou, China) equipped with a Windows analysis platform and a multi exponential fitting analysis (T-invfit) program. The evaluative method was modified according to a previous study [51]. Briefly, colloidal particles suspension were collected every 20 min during the heating process, subsequently about 10 mL of HIPEs were prepared. Water migration of the HIPEs was determined and the test conditions were set up as follows: the ambient temperature was 32 °C and the proton resonance frequency was 22 MHz corresponded to the strength of the magic field of 0.53 T. Approximately 10 mL of the samples were placed in a 25 mm diameter LF-NMR tube and then placed in the analyzer. The time between the 90° pulse and the 180° pulse was 250 μs. Actually, about 10,000 echoes were obtained after 16 repeated scans with a repetition interval of 6.52 s. The obtained graph was an exponential decay graph with 4 replicates for each test. The transverse relaxation (T_2_) was measured using a Carr-Purcell-Meiboom-Gill pulse sequence (CPMG), and the time corresponding to the peak value of each component in the relaxation diagram was taken as T_2_.

#### 4.5.3. CLSM

Confocal microscope (Leica TCS SP8, Leica Microsystems Inc., Heidelberg, Germany) was used for CLSM analysis according to the method described by [56] with slight modifications. In brief, 1 mL of emulsion was mixed with 20 μL of 0.1% (*w*/*v*) Nile Red and 10 mg/mL Fast Green for staining for 1 h. An appropriate amount of stained samples were applied to glass slides, covered with the coverslip, and placed upside down on the loading table for observation. The excitation wavelength was set at 488 nm and 633 nm, and the wavelength at 488 nm was used to detect Nile Red (stained oil) and the wavelength at 633 nm was used to detect Fast Green (stained protein). Photos taken by confocal microscope were processed using LAS AF Lite 2.3.5 (Leica Microsystems Inc., Heidelberg, Germany).

### 4.6. Statistical Analysis

The data were processed and reported as mean ± standard deviation. A one-way analysis of variance was performed at the probability level of 0.05 according to Duncan’s multiple range tests. Statistical analysis was carried out using SAS 8.0 software (SAS version 9.0, SAS Institute, Cary, NC, USA).

## Figures and Tables

**Table 1:** Formulations of Pickering emulsions.

Samples	Ultrasound Modes	Ultrasonic Frequency (Hz)
20	SFU	20
40		40
60		60
20/40	DFU	20/40
20/60		20/60
40/60		40/60
24/40/60	MFU	20/40/60

**Table 2:** The zeta-potential, PDI, hydrodynamic radius of samples (zein: the colloidal particles prepared with native zein; zein + DFU: the colloidal particles prepared with zein treated with DFU; zein + DFU + SSPS: the colloidal particles prepared with DFU combined with SSPS).

Samples	Zeta-Potential	PDI (%)	Hydrodynamic Radius
Zein	18.48 ± 0.23 b	15.70 ± 0.95 b	132.23 ± 0.85 b
Zein + DFU	18.33 ± 1.00 b	11.30 ± 1.15 a	114.54 ± 0.23 a
Zein + DFU + SPSS	−21.90 ± 0.46 a	17.47 ± 0.90 b	256.5 ±4.81 c

Values are mean ± SD from triplicate determinations. Means with different letters in same column are significantly different (*p* < 0.05).

## Data Availability

The data presented in this study are available in the article.
